# Adiponectin inhibits neutrophil apoptosis via activation of AMP kinase, PKB and ERK 1/2 MAP kinase

**DOI:** 10.1007/s10495-013-0893-8

**Published:** 2013-08-28

**Authors:** Alessandra Rossi, Janet M. Lord

**Affiliations:** MRC Centre for Immune Regulation, School of Immunity and Infection, University of Birmingham, Birmingham, B15 2TT UK

**Keywords:** Adiponectin, Neutrophils, Apoptosis, Mcl-1, AMPK

## Abstract

Neutrophils are abundant, short-lived leukocytes that play a key role in the immune defense against microbial infections. These cells die by apoptosis following activation and uptake of microbes and will also enter apoptosis spontaneously at the end of their lifespan if they do not encounter a pathogen. Adiponectin exerts anti-inflammatory effects on neutrophil antimicrobial functions, but whether this abundant adipokine influences neutrophil apoptosis is unknown. Here we report that adiponectin in the physiological range (1–10 μg/ml) reduced apoptosis in resting neutrophils, decreasing caspase-3 cleavage and maintaining Mcl-1 expression by stabilizing this anti-apoptotic protein. We show that adiponectin induced phosphorylation of AMP-activated kinase (AMPK), protein kinase B (PKB), extracellular signal-regulated kinase (ERK 1/2) and p38 mitogen activated protein kinase (MAPK). Pharmacological inhibition of AMPK, PKB and ERK 1/2 ablated the pro-survival effects of adiponectin and treatment of neutrophils with an AMPK specific activator (AICAR) and AMPK inhibitor (compound C) respectively decreased and increased apoptosis. Finally, activation of AMPK by AICAR or adiponectin also decreased ceramide accumulation in the neutrophil cell membrane, a process involved in the early stages of spontaneous apoptosis, giving another possible mechanism downstream of AMPK activation for the inhibition of neutrophil apoptosis.

## Introduction

Neutrophils, the most numerous immune cells in the circulation, represent the first line of protection against microbial and fungal infection. These post mitotic cells are characterised by a very short lifespan, undergoing constitutive apoptosis within 5 days after leaving the bone marrow [1]. However their apoptosis can be delayed at sites of infection by a range of factors including bacterial products such as lipopolysaccharide (LPS) [2], pro-inflammatory cytokines [3, 4] and hypoxia [5], ostensibly to extend their functional lifespan. However, the tight regulation of apoptosis and the prompt removal of apoptotic neutrophils are also central to the resolution of the inflammatory response and prevention of tissue damage and chronic inflammation [6, 7].

Neutrophils express very few Bcl-2 family members, with Mcl-1 appearing to be the most relevant in regulating their survival [8]. The level of this protein positively correlates with neutrophil lifespan [9] and its loss accelerates neutrophil apoptosis [10, 11]. Due to its characteristic PEST domain, this protein is subject to rapid turnover by the proteasome [12], however the activation of several signalling pathways can modulate Mcl-1 levels therefore influencing neutrophil longevity. In particular, the activation of the MAPK ERK 1/2 and the PI3K/PKB axis following treatment of cells with LPS [13], TLR agonists [14] and granulocyte macrophage colony-stimulating factor (GM-CSF) [15], results in the maintenance of Mcl-1. Both ERK 1/2 and PI3K/PKB have been found to increase Mcl-1 protein levels mainly by stabilizing the protein through specific phosphorylations which delay Mcl-1 degradation and increase its half-life [16].

p38 MAPK is also phosphorylated in response to LPS [13] and hypoxia [5, 17], though its anti-apoptotic effect is less clear as its pharmacological inhibition further increases LPS anti-apoptotic effects [13]. More recently we have shown that inhibition of cyclin dependent kinase (CDK) 9 results in loss of Mcl-1 [18], identifying the cell cycle independent CDKs as novel regulators of neutrophil apoptosis. Additional proteins such as survivin [19] and proliferating cell nuclear antigen [20] have been proposed as regulators of neutrophil lifespan, as they are present in mature neutrophils and their expression correlates with decreased apoptosis.

Adiponectin is an abundant adipokine which belongs to the C1q/tumor necrosis factor superfamily [21, 22]. The full-length isoform displays anti-inflammatory properties on neutrophils as it inhibits superoxide generation [23] and production of the chemokine CXCL8 [24]. The main signaling pathway activated by adiponectin and responsible for its actions in several cell types is proposed to be AMPK [25]. To date there are no reports of a role of adiponectin or AMPK in regulating neutrophil apoptosis. In this study we show that adiponectin delayed apoptosis of human neutrophils by activating AMPK, PKB, ERK 1/2, revealing for the first time that signalling through AMPK enhances neutrophil lifespan and is required for adiponectin’s anti-apoptotic effect.

## Materials and methods

### Reagents and antibodies

Human recombinant adiponectin, was purchased from Enzo Life Sciences (Farmingdale, NY), with contamination from LPS certified to be less than 0.1 EU/μg purified protein. To ensure no artefactual effects from LPS contamination, Polymyxin B (10 μg/ml) (Millipore, Billerica, MA) was added to the cells 30 min before treatment with adiponectin. Percoll, RPMI 1640 medium, l-glutamine, penicillin–streptomycin, propidium iodide solution (PI), dimethylsulfoxide (DMSO), fetal calf serum (FCS), goat serum, cycloheximide, protease inhibitor cocktail (catalogue number P8348) and phosphatase inhibitor cocktails (catalogue numbers P0044 and P5726), the AMPK inhibitor compound C (6-[4-(2-Piperidin-1-ylethoxy)phenyl]-3-pyridin-4-ylpyrazolo[1,5-a]pyrimidine) and the p38 inhibitor SB202190 and all buffers and salt solutions were purchased from Sigma-Aldrich (Poole, UK). The MEK1 inhibitor PD98059 was from Cell Signaling Technology (Beverly, MA), the PI3K inhibitor LY294002 was from Millipore, Annexin V-FITC was obtained from BD Biosciences (San Jose, CA), the AMPK activator AICAR (5-Aminoimidazole-4-carboxamide 1-β-D-ribofuranoside) and the anti-ceramide antibody (clone MID15B4) were from Enzo Life Sciences. FITC-conjugated anti-active caspase-3 antibody was purchased from BD Biosciences (San Jose, CA). Antibodies against phosphorylated AMPK (Thr172), total AMPK, phosphorylated PKB (Ser473), total PKB, phosphorylated ERK1/2 (Thr202/Tyr204), total ERK and phosphorylated p38 MAPK (Thr180/Tyr182), were all purchased from Cell Signaling Technology. The antibodies against total p38 MAPK and Mcl-1 (clone S-19) were from Santa Cruz Biotechnology (Santa Cruz, CA) and the antibody against actin was obtained from Sigma-Aldrich. The secondary antibody FITC-labelled goat anti-mouse was obtained from Southern Biotech (Birmingham, AL) and the isotype control mouse IgM was from Dako (Ely, UK).

### Neutrophil isolation and treatment

Heparinised peripheral blood was obtained from healthy human donors and all volunteers gave written informed consent prior to their participation. The study was approved by the local research ethics committee. Neutrophils were isolated by density centrifugation as previously described [26]. The purity of isolated neutrophils was determined by differential staining using a commercial May-Grunwald Giemsa stain (Diff-Quick, Baxter Healthcare, UK) and light microscopy and was routinely greater than 97 %. For all studies neutrophils were resuspended in RPMI-1640 medium containing 2 Mm l-glutamine, 100 U/ml penicillin, 100 μg/ml streptomycin supplemented with 10 % heat-inactivated FCS.

Isolated neutrophils were adjusted to a concentration of 2 × 10^6^/ml and were dispensed into a 96-well round bottomed plate (Sarstedt, Leicester, UK) and incubated with either adiponectin at the indicated doses or vehicle (sterile distilled water) for the indicated incubation times. The kinase inhibitors compound C, PD98059 and SB202190 were added to the culture 30 min before the addition of adiponectin at a concentration of 10 μM. Cycloheximide was added 30 min before the addition of adiponectin at the final concentration of 5 μg/ml. Compound C (10 μM) and AICAR (1 mM) were also added to purified neutrophils for 20 h to determine whether specific AMPK activation/inhibition affected neutrophil apoptosis.

### Assessment of neutrophil apoptosis

Apoptosis was determined by three methods. For Annexin V binding, which identifies cells with phosphatidylserine exposed on the cell surface, cells were washed with PBS and centrifuged at 250×*g* for 5 min at 4 °C, then resuspended in 100 μl of Annexin V buffer (10 mM HEPES, pH 7.4; 140 mM NaCl; 2.5 mM CaCl_2_). 1 μl of AnnexinV-FITC was added per well (1 × 10^5^ cells) and cells were incubated in the dark at room temperature for 10 min, after which PI was added at a final concentration of 5 μg/ml. Fluorescence was analyzed by flow cytometry (BD Accuri C6 Flow Cytometer, Accuri Cytometers Inc., Ann Arbor, MI), with cells that were Annexin V positive but PI negative taken as apoptotic.

Apoptosis was also measured by assessment of cells with the nuclear morphology characteristic of apoptotic neutrophils, i.e. collapse of the multi-lobed nucleus to a single densely stained body. Cytospin preparations were differentially stained using a commercial May-Grunwald Giemsa stain and assessed for apoptotic morphology by light microscopy using an Olympus IX71 microscope. Images were taken at 40× magnification.

Apoptotic neutrophils express active caspase 3. To detect active caspase 3 cells were fixed and permeabilised using the Fix and Perm^®^ kit from Life technologies (Carlsbad, CA), according to the manufacturer’s instructions. Cells were washed and resuspended in 100 μl of PBS, then FITC-conjugated anti-active caspase-3 or isotype matched antibody were added to a final concentration of 2.5 μg/ml and incubated for 30 min at room temperature. Neutrophils were washed and immunofluorescence analyzed by flow cytometry.

### Protein extraction and western blotting

Neutrophils were spun at 4,000 rpm for 4 min (MSE microcentaur) prior to resuspension in lysis buffer (20 mM MOPS, 50 mM NaF, 50 mM β-glycerophosphate, 50 mM Na_3_VO_4_, 1 % Triton X-100, 1 mM DTT, 1 mM AEBSF and 1 % protease [27] and phosphatase inhibitor cocktails [28]). Lysis of neutrophils was performed on ice for 30 min with occasional vortexing. The lysates were centrifuged at 13,000 rpm for 1 min (MSE microcentaur) and the supernatant collected and combined with an equal volume of SDS-PAGE sample buffer (125 mM HCl pH 6.8, 5 % glycerol, 2 % SDS, 1 % β-mercaptoethanol, 0.003 % bromophenol blue) and boiled for 10 min. Proteins were separated by SDS-PAGE and transferred onto PVDF membranes. Non-specific protein binding was blocked using either 5 % BSA for the phosphospecific antibodies or 5 % non-fat milk for the remaining antibodies. Membranes were incubated with primary antibodies to phosphorylated AMPK (1:1,000), total AMPK (1:1,000), phosphorylated PKB (1:1,000), total PKB (1:1,000), phosphorylated ERK 1/2 (1:1,000), total ERK (1:1,000), phosphorylated p38 MAPK (1:1,000), total p38 MAPK (1:500), Mcl-1 (1:1,000), or β-actin (1:5,000) overnight at 4 °C. After washing, membranes were incubated with appropriate secondary antibodies (ECL™ anti-rabbit or anti-mouse IgG; GE Healthcare, Uppsala, Sweden) for 1 h at room temperature. Proteins were visualized by ECL according to manufacturer’s instructions (GeneFlow, Lichfield, UK). When necessary, membranes were subjected to mild stripping (stripping buffer: 200 mM glycine, 0,1 % SDS, 1 % Tween 20). Densitometric analyses were performed using Image J software.

### Surface detection of ceramide

Surface accumulation of ceramide was analyzed by indirect immunofluorescence staining after 20 h of treatment. Briefly, neutrophils were washed with PBS and monoclonal antibody against ceramide was added at a final concentration of 10 μg/ml and cells were incubated for 30 min at 4 °C. After the incubation cells were washed with PBS and spun at 250×*g* for 5 min at 4 °C, then resuspended in PBS. Nonspecific binding sites were blocked by addition of 3 μl of goat serum for 5 min before addition of the secondary FITC-conjugated goat anti-mouse antibody (10 μg/ml) for a further 30 min. Samples were washed with PBS and analyzed by flow cytometry.

### Statistical analyses

Data were analyzed using GraphPad Prism 4 software (GraphPad Software Ltd, La Jolla, CA). Two-tailed paired Student’s *t* test was used to compare two groups of paired samples, whereas repeated measures ANOVA was used to analyze more than two groups of matched samples, followed by Tukey’s multiple comparison test. Results are expressed as mean values ± standard error of the mean (SEM). A *p* value of less than 0.05 was accepted as significant.

## Results

### Adiponectin inhibits neutrophil apoptosis

**Fig. 1:**
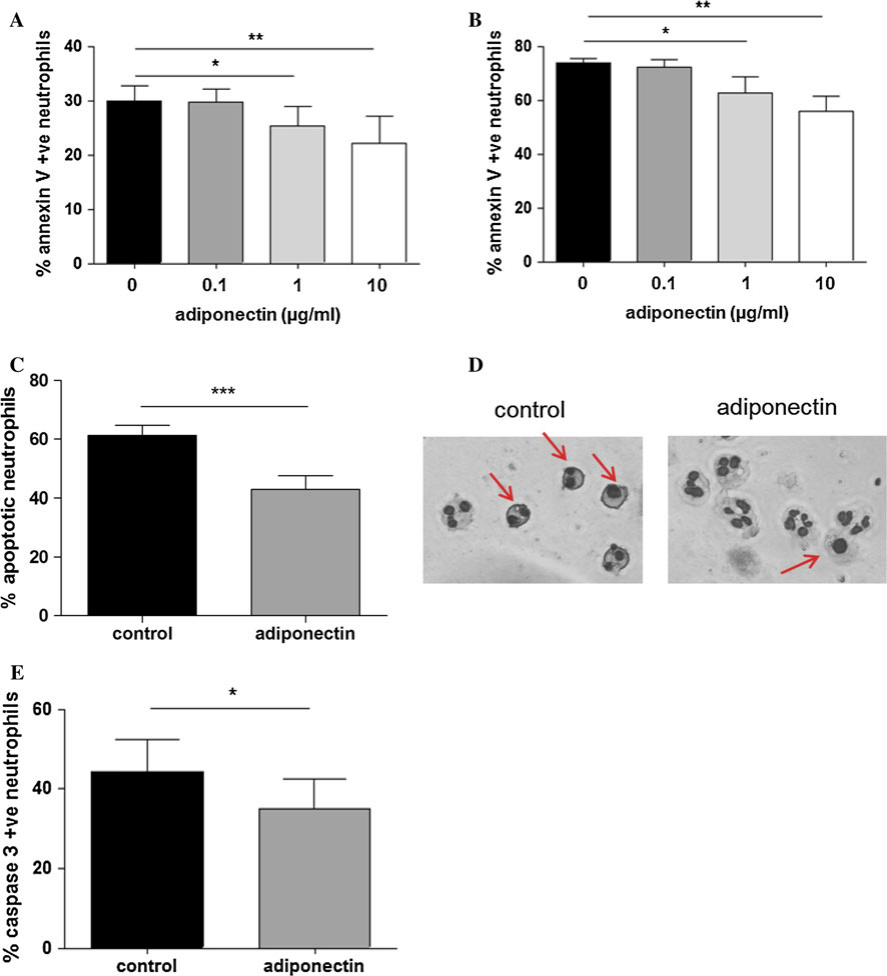
Adiponectin delays neutrophil spontaneous apoptosis. Human neutrophils were treated with adiponectin at the concentratiosn shown and assessed for the presence of apoptotic cells by staining for Annexin V binding at **a** 6 h and **b** 20 h incubation (*n* ≥ 6 experiments); **c** Apoptosis was also assessed after 20 h by examination of differentially stained cells for nuclear morphology characeristic of apoptotic neutrophils (*n* = 5 experiments) and **d** representative images are shown: *arrows* indicate late apoptotic neutrophils; **e** The presence of active caspase 3 was also assessed by immunostaining. In **a**, **b**, **c** and **e** data are mean ± SEM and *p < 0.05 and **p < 0.01

We found that addition of adiponectin in the physiological range (1–10 μg/ml [29, 30]) reduced neutrophil apoptosis in a dose dependent manner after both 6 and 20 h incubation (Fig. 1a, b). At a higher concentration (20 μg/ml) adiponectin did not further increase the rescue from apoptosis (data not shown). Therefore, 10 μg/ml adiponectin was used in the remaining experiments. Examination of the nuclear morphology of neutrophils confirmed the anti-apoptotic effect of adiponectin. After 20 h of incubation, control neutrophils displayed fragmented or mono-lobed nuclei (indicated with arrows) whereas a greater number of adiponectin-treated neutrophils retained the multi-lobed nuclear morphology of healthy, non-apoptotic cells (Fig. 1c, d). Caspase-3 represents a central element in neutrophil constitutive apoptosis as its activation induces the characteristic changes in the apoptotic cell, including the final DNA fragmentation [31]. To assess whether adiponectin inhibited neutrophil apoptosis through decreased activation of caspase-3, we stained neutrophils with an antibody against its cleaved active form. As shown in Fig. 1e, after 20 h adiponectin significantly decreased the percentage of cells displaying active caspase-3.

### Adiponectin treatment prevents loss of Mcl-1 expression

**Fig. 2:**
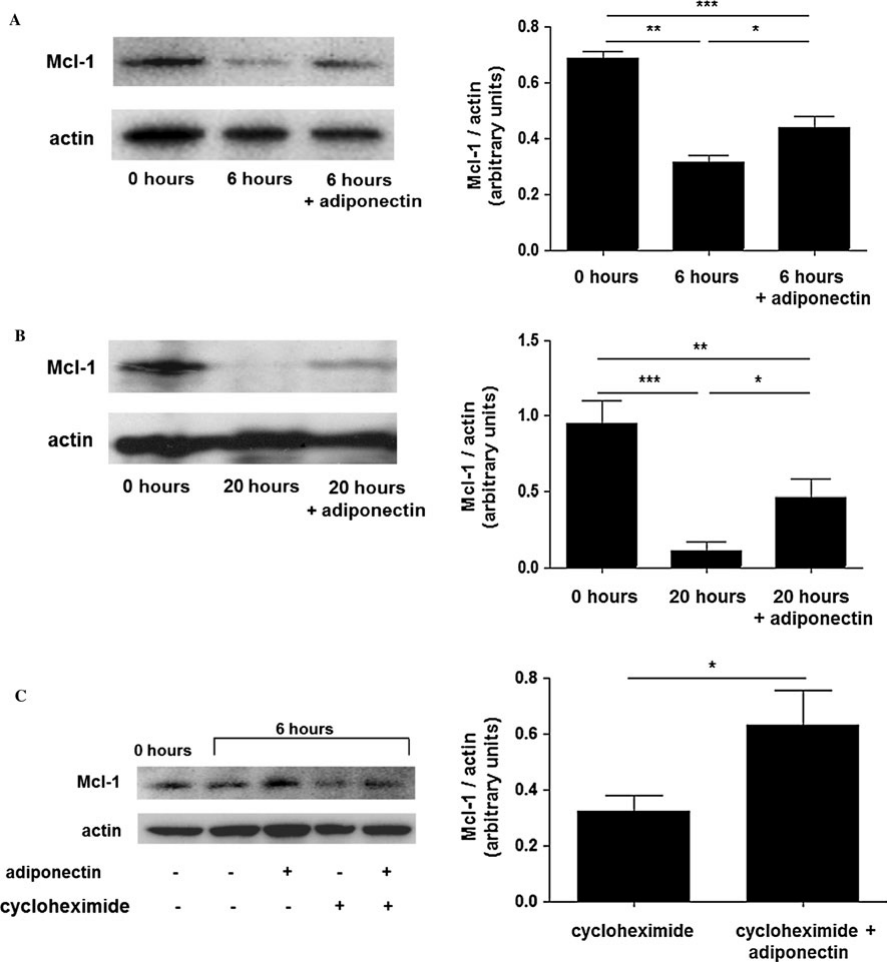
Adiponectin maintains Mcl-1 protein levels by increasing Mcl-1 protein stability. Representative western blots and densitometric analyses showing the changes in the level of Mcl-1 in freshly isolated, untreated and adiponectin-treated neutrophils after 6 h (**a**) and 20 h (**b**) of incubation. **c** Representative western blots and densitometric analysis showing the effect of cycloheximide (5 μg/ml) on the adiponectin-mediated increase in Mcl-1. Densitometric analyses are expressed as the ratio of Mcl-1 to β-actin. Data are mean ± SEM and *p < 0.05, **p < 0.01, ***p < 0.001

Mcl-1 is the Bcl-2 family protein most involved in regulating neutrophil apoptosis [9] and its expression is increased by several anti-apoptotic stimuli [16]. Thus, we evaluated whether adiponectin-mediated inhibition of neutrophil apoptosis was associated with upregulation of Mcl-1. As shown in Fig. 2a, b, adiponectin-treated neutrophils exhibited higher expression of Mcl-1 than untreated cells after both 6 and 20 h of incubation. To determine whether this was achieved through improved stability of Mcl-1, we pre-treated the cells with the protein synthesis inhibitor cycloheximide (5 μg/ml) and assessed Mcl-1 levels after 6 h of incubation. As previously reported [16], we found that cycloheximide treatment alone reduced Mcl-1 protein levels and we observed that addition of adiponectin to cycloheximide-treated cells resulted in an increase in Mcl-1 levels, confirming that adiponectin decreased the turnover of Mcl-1 (Fig. 2c).

### Adiponectin activates AMPK, PKB and the MAPKs ERK 1/2 and p38

**Fig. 3:**
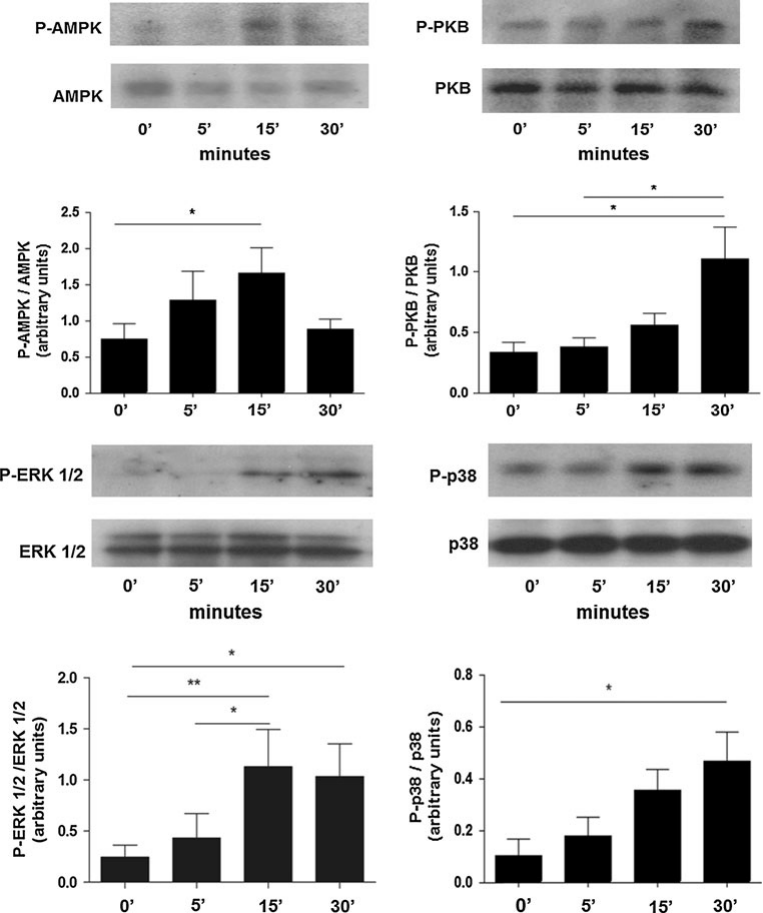
Adiponectin induces phosphorylation of AMPK, PKB, ERK 1/2 and p38 MAPK. Representative western blots and densitometric analyses showing time courses for AMPK, PKB, ERK 1/2 and p38 phosphorylation induced by adiponectin. Densitometric analyses are expressed as the ratio of phosphorylated to unphosphorylated forms of the proteins (*n* = 4). Data are mean ± SEM and *p < 0.05, **p < 0.01, ***p < 0.001

The main intracellular signalling pathways previously reported to be activated by adiponectin are AMPK and p38 MAPK [23, 32]. We investigated whether adiponectin could promote the phosphorylation of these two kinases, plus other kinases known to modulate neutrophil apoptosis, namely the PI3K substrate PKB and ERK 1/2 MAPK [13, 14, 33]. Studies of the time course of kinase activation showed that adiponectin transiently activated AMPK for up to 15 min (Fig. 3). Adiponectin also induced the phosphorylation of PKB, ERK 1/2 and p38 MAPKs, though the kinetics of activation were different and were maximal at 30 min of adiponectin treatment (Fig. 3).

### AMPK, PKB and ERK 1/2 inhibition block the anti-apoptotic effect of adiponectin

**Fig. 4:**
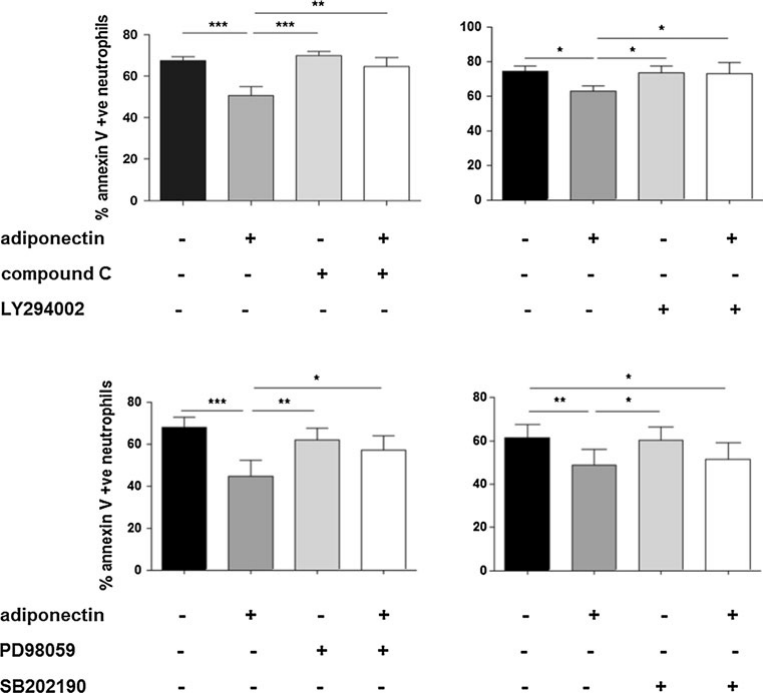
Anti-apoptotic effects of adiponectin are mediated by AMPK, PKB and ERK 1/2 activation. Neutrophils were incubated with pharmacological inhibitors of AMPK (compound C, 10 μM), PI3K/PKB (LY294002, 10 μM), ERK MAPK (PD98059, 10 μM), or p38 MAPK (SB202190, 10 μM) prior to treatment with adiponectin (10 μg/ml). Apoptosis was measured by Annexin-V/PI staining after 20 h of incubation (*n* = 7). Data are mean ± SEM and *p < 0.05, **p < 0.01, ***p < 0.001

To assess whether the activation of AMPK, PKB, ERK 1/2 and p38 MAPK were all required for adiponectin’s anti-apoptotic effects, we used selective pharmacological inhibitors against these kinases prior to the addition of adiponectin and monitored neutrophil apoptosis by Annexin V/PI staining after 20 h. The data show that the adiponectin-mediated increase in neutrophil survival was blocked by inhibition of AMPK, PI3K/PKB and ERK 1/2, but not p38 MAPK (Fig. 4).

**Fig. 5:**
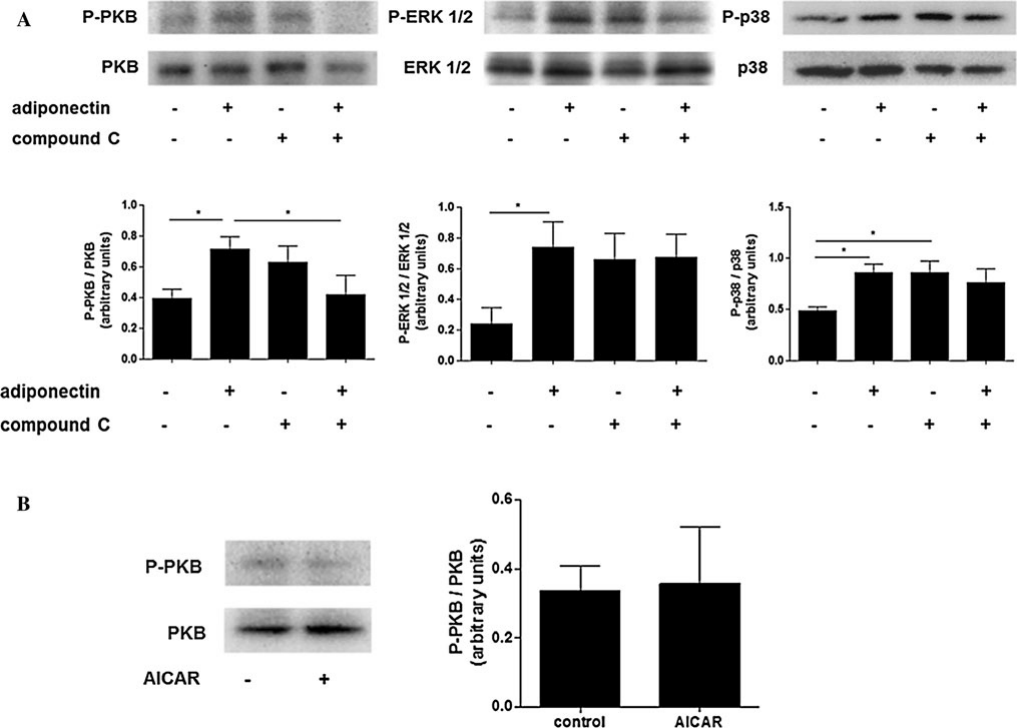
Adiponectin may not activate PKB, ERK 1/2 and p38 via AMPK. Representative western blots and densitometric analyses showing **a** phosphorylation of PKB, p 42/44 ERK and p38 in response to treatment of neutrophils with adiponectin (10 μg/ml) in the absence or presence of compound C (10 μM) (*n* = 6) and **b** effect of AICAR treatment (1 mM) on PKB phosphorylation (*n* ≥ 3). Densitometric analyses are expressed as the ratio of the phosphorylated to unphosphorylated forms of the proteins. Data are mean ± SEM and *p < 0.05, **p < 0.01, ***p < 0.001

As the phosphorylation of AMPK induced by adiponectin preceded the phosphorylation of PKB, ERK 1/2 and p38 (Fig. 3), it was important to investigate the role of AMPK in adiponectin-mediated activation of these signalling pathways. We therefore pre-incubated neutrophils with the AMPK inhibitor compound C for 30 min prior to addition of adiponectin for a further 30 min and assessment of the phosphorylation of PKB, ERK 1/2 and p38 MAPK. Compound C on its own led to an increase in the phosphorylation of PKB, ERK 1/2 and p38 compared to the control although this only reached statistical significance for phosphorylation of p38. Importantly, AMPK inhibition by compound C led to complete inhibition of adiponectin induced phosphorylation of PKB, with no significant effect on ERK or p38, suggesting that AMPK activation could be upstream of PKB activation (Fig. 5a). We then stimulated neutrophils with the pharmacological activator of AMPK, AICAR (1 mM) for 30 min, but AICAR had no effect on the phosphorylation of PKB (Fig 5b) or ERK 1/2 and p38 phosphorylation (data not shown). From these data we cannot conclusively conclude that AMPK activation by adiponectin lies upstream of PKB activation.

### AMPK activation is a survival signal for neutrophils

**Fig. 6:**
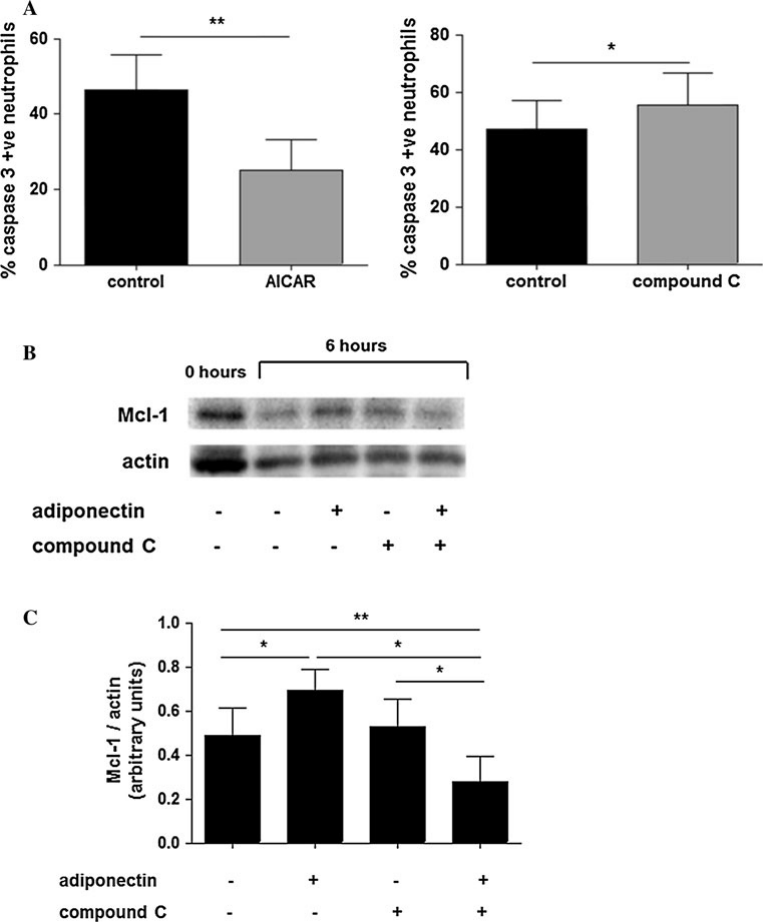
Pharmacological AMPK activation and inhibition modulates neutrophil apoptosis. Neutrophils were incubated with the AMPK activator AICAR (1 mM) or inhibitor compound C (10 μM). **a** Apoptosis was measured by caspase-3 activation after 20 h incubation; **b** representative western blots and **c** densitometric analysis showing the effect of compound C and adiponectin on Mcl-1 levels after 6 h treatment. Densitometric analyses are expressed as the ratio of Mcl-1 to β-actin (*n* = 6). Data are mean ± SEM and *p < 0.05, **p < 0.01

AMPK activation is triggered by low intracellular ATP levels [34] and it usually induces apoptosis in cancer cells [35] but enhances survival in other post mitotic cells, such as neurons [36]. To further investigate the role of AMPK itself in neutrophil apoptosis, we evaluated the percentage of apoptotic cells in the presence of the pharmacological AMPK activator AICAR and its inhibitor compound C. AMPK activation decreased neutrophil apoptosis as assessed by caspase-3 activation after 20 h incubation (Fig. 6a), but did not affect Mcl-1 levels (data not shown). Although treatment with compound C alone gave a slight but non-significant increase in apoptosis as measured by Annexin V staining (Fig. 4), when apoptosis was measured by caspase 3 activation the increase reached significance (Fig. 6a). Mcl-1 levels were decreased by addition of compound C in adiponectin-treated cells, but were not significantly affected by compound C on its own (Fig. 6b, c).

### Adiponectin, AICAR and compound C alter ceramide accumulation in the membrane

**Fig. 7:**
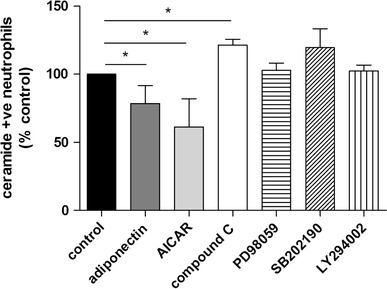
Adiponectin and modulators of AMPK activity influence the cell membrane accumulation of ceramide. Neutrophils were incubated for 20 h with adiponectin (10 μg/ml), AICAR (1 mM), compound C (10 μM), PD98059 (10 μM), SB202190 (10 μM) and LY294002 (10 μM) (*n* ≥ 4) and the surface level of ceramide was determined. Data are expressed as mean percentage relative to control ± SEM and *p < 0.05

Ceramide is a sphingolipid which physiologically accumulates in neutrophils inducing their apoptosis [37, 38], in particular its assembly in membrane lipid rafts accelerates death receptor-induced cell death [38, 39]. Ceramide generation has already been demonstrated to be regulated by AICAR [40], compound C [41] and adiponectin itself [42], hence we analysed the content of ceramide present on the surface of neutrophils after 20 h of incubation with these compounds. We also assessed ceramide accumulation in the presence of inhibitors of PI3K, ERK 1/2 and p38 MAPKs to determine whether the signaling pathways activated by adiponectin could contribute to altered ceramide accumulation (Fig. 7). AICAR and compound C respectively decreased and increased ceramide levels on neutrophil membranes, indicating the possible mechanism by which AMPK activation could influence neutrophil apoptosis. Adiponectin also significantly diminished ceramide accumulation, although its effect was less marked than AICAR treatment (Fig. 7). In contrast, PI3K, ERK 1/2 and p38 inhibition did not affect ceramide content (Fig. 7).

## Discussion

Neutrophil constitutive apoptosis can be modulated by a range of factors which interfere with pro- and anti-apoptotic signalling pathways, resulting in extended or shortened lifespan [43]. Our findings reveal that adiponectin is another systemic factor that negatively influences neutrophil apoptosis in vitro. Moreover our data suggest that this is mediated by mechanisms that include stabilisation of Mcl-1 and reduced accumulation of cermide in the cell membrane, the latter mediated by AMPK activation.

Adiponectin has been extensively studied with regard to its anti-inflammatory actions [29], although some groups have also described pro-inflammatory effects, for example on dendritic cells [44], macrophages and lymphocytes [32]. Contradictory results exist in relation to its effect on apoptosis: adiponectin induces apoptosis in several tumor cell lines [45–47] as well as activated T lymphocytes [48], whereas it protects post-mitotic cell types from death, such as neurons [49], and endothelial cells [50] when challenged with pro-apoptotic agents. Moreover, an association between low serum adiponectin concentration and high levels of apoptotic markers in the blood was reported recently [24]. Taking into account this literature, the anti-apoptotic effect indicated here for adiponectin on neutrophils, a post-mitotic cell, is consistent with the previous findings for its effects on non-proliferating cells.

Whether inhibition of neutrophil apoptosis would be pro- or anti-inflammatory in vivo remains to be established. At times of infection extending neutrophil lifespan could be beneficial as it would aid removal of the infective agent, but as others have reported inhibition of neutrophil functions such as superoxide generation [23] by adiponectin, keeping these cells alive but non-functional could be pro-inflammatory. This conclusion is supported clinically as adiponectin levels are higher in certain chronic inflammatory diseases, such as chronic obstructive pulmonary disease (COPD) [51], in which reduced levels of neutrophil apoptosis are well documented [52, 53].

This study shows that adiponectin enhances neutrophil survival by decreasing the cleavage of caspase-3 and reducing Mcl-1 degradation and accumulation of ceramide in the cell membrane. The main anti-apoptotic signalling pathways activated by adiponectin were shown to be AMPK, PI3K/PKB and the MAPK ERK 1/2. The latter two pathways are already known to increase Mcl-1 half-life and thereby reduce neutrophil apoptosis [16]. Phosphorylation of p38 was also induced by adiponectin and this pathway has been shown to enhance NF-kB activity, which is known to inhibit neutrophil apoptosis [54, 55], either by phosphorylation of RelA/p65 [56] or by promoting IKKb degradation [57]. Adiponectin itself has been reported to activate NF-kB under certain conditions [58, 59]. However here we show that p38 inhibition did not block the anti-apoptotic effects of adiponectin.

Our data also suggest that AMPK activation was not responsible directly for the adiponectin-mediated phosphorylation of PKB, ERK 1/2 and p38 as the pharmacological AMPK activator AICAR did not induce activation of these pathways. Although the addition of the AMPK inhibitor compound C inhibited adiponectin-mediated phosphorylation PKB, it also led to an increase in p38 phosphorylation and a slight but non significant effect on ERK and PKB when added to cells in the absence of adiponectin. These indicate that compound C may have off target effects that led to activation of these kinases and compound C has been reported to exert effects independent of AMPK activation by others [60–62]. Therefore we cannot conclude with certainty that activation of PKB, ERK1/2 and p38 occur downstream of AMPK activation and the AICAR data certainly do not support this.

To further investigate the role of AMPK activity in regulating neutrophil apoptosis we demonstrated that the activating agent AICAR reduced neutrophil apoptosis without increasing Mcl-1 stabilization. However, the literature contains reports that both AICAR and compound C have effects upon the generation of ceramide within the cell membrane, through modulation of sphingomyelinase [40, 41]. We confirmed these results in neutrophils and also showed that adiponectin significantly decreased the accumulation of ceramide in neutrophil cell membranes. These findings are potentially important as we have previously shown a key role for ceramide generation in triggering lipid raft coalescence and activation of death receptor signalling in neutrophils during constitutive apoptosis [37]. Importantly, inhibition of PI3K, ERK 1/2 and p38 did not influence ceramide accumulation, suggesting that AMPK is the only modulator of ceramide level among the pathways activated by adiponectin.

In conclusion, our work demonstrates that adiponectin exerts an anti-apoptotic effect on neutrophils mainly by activating AMPK, PI3K/PKB and ERK pathways and preliminary data suggest that both stabilisation of Mcl-1 and reduced ceramide accumulation in the cell membrane are involved.
